# Euthanasia*Written for the Kansas Homœopathic Medical Society, 1894.

**Published:** 1894-06

**Authors:** W. A. Yingling

**Affiliations:** Nonchalanta, Kan.


### EUTHANASIA.*

W. A. YINGLING, M. D., NONCHALANTA, KAN.

Mrs. S. K., aged sixty-nine; mother of six children. She had been feeling unwell and very feeble for several months. An asthmatic subject. Has had the pneumonia several times, typhoid fever, and severe spells of acute rheumatism.

Last Tuesday by stepping to the yard to bring in coal the wind, not very cold, brought on congestion of the lungs, devel-

* Written for the Kansas Homœopathic Medical Society, 1894.

oping into pleuro-pneumonia of the left side. The allopathic attendant fed her on the usual palliatives and large doses of the physician's shame, Morphine.

At 8 P. M. on the evening of March 9th, 1894, I found her completely under the influence of Morphine, unconscious, very labored and loud breathing, tossing about with quite frequent moaning, a quick movement of the hand to the side, distortion of the face and a piteous cry indicative of severe pain. Very weak; propped up in bed; could not lie down. No pulse at wrist perceptible.

I could find no indications for a remedy with such a complicated drug disease, but as she was so weak and death seemed so very imminent I placed on her tongue a small powder of *China*$^{54m}$ (Deschere), and had to wet the tongue with water as it was so extremely dry. The China seemed to rally her in a very short time.

A while after midnight her lungs filled up with mucus, without expectoration, but with great rattling; cold nose; tongue and mouth very dry and parched; throat externally swollen even with the chin and seemed to be broadened as well as extended; very acute pain, with moaning; could not swallow at all; rapid, loud breathing; the left side seemed easier by pressure of the hand and a very hot water-bag; had to sit nearly upright and be supported in bed. Unable to speak and therefore to give her symptoms. All indications pointed to immediate dissolution. Friends were called, and every one looked for the end to be each moment. It seemed that she could not but be choked to death by the great accummulation of mucus in the throat and upper part of the lungs, yet there was no power of expectoration.

I placed a dry powder of *Antimony-tart.*$^{cm}$ (Swan), on the tongue and moistened it with a few drops of water. In a very brief time there was a change for the better, with gradual improvement during the night and the following morning. The phlegm almost entirely disappeared and gave no trouble at all in breathing. The swelling of the throat went down rapidly, and she seemed quite comfortable.

About noon of the 10th the phlegm again collected in the throat, with a tendency to a repetition of the previous condition. Being thirty-five miles from my office I was compelled to use the same potency, and so placed a small powder of the same remedy on her tongue, moistening it as before with a little water. A very speedy relief followed.

Continued to gradually gain intelligence, with more natural breathing. Acute pain occasionally, but not so frequent nor so severe. Still unable to give her symptoms. At midnight following she became very restless with tossing about, throwing the hands; moaning; very little rattling of mucus in the throat; greedily taking water from the finger or cloth; great anxiety. Placed a powder of *Arsenicum-alb.*$^{cm}$ (H. S.), on the tongue dry. Very soon, so soon as to be remarked by the friends, there was quiet and more natural sleep.

March 11th I made this note, which I give as written at the bedside: "This A. M. she seems very quiet, but very weak. Tongue more moist. Has more consciousness and can readily recognize her children, who have come to see her. Pulse at wrist now perceptible. Can lie down flat in bed. There is no moaning, nor crying out from pain. Pain seems to have subsided. Eyes are not glassy as they have been. Sleeps more natural and easy; with the exception of the great weakness and her age she would be in a fair way to recovery. Begins to enjoy a little buttermilk and weak tea, which she can now swallow. One P. M.—Has been resting easy, but spells of disquiet; a very little rattling of phlegm in the lungs; eyes glazed and sight very feeble; gradually, but surely sinking. But very little indication of pain."

Between three and four o'clock of the afternoon (11th), the fever began to rise, which was the usual time; restless with some tossing about. Breathing more labored and shorter, quite audible. Growing weaker all the time. Occasional attempts to cough, but seems too weak. Gave *Arsenicum-alb.*$^{cm}$ (H. S.), in water, a spoonful every hour till a change for the better; three doses given.

Six P. M.—Resting easy, but breathing rapid and audibly; somewhat restless; no apparent suffering.

March 12th.—Rested easy during the night, but breathing short and labored; no perceptible suffering; eyes more sunken; weaker and gradually sinking; can live but a couple hours.

At nine A. M. (12th) she died, without a struggle, without pain, and with great quietness. "There was a gush of bloody water from the mouth, and then she simply ceased to breathe."

I report this case to prove that the homœopathic remedy in the potentized form can and does produce euthanasia even when the allopathic practice of huge doses of Morphine entirely failed. Morphine surely acts as efficiently in the hands of an allopath as it does in the hands of a homœopath. In this instance Morphine failed to give any relief at all, but aggravated the case in every way. The homœopathic remedy relieved all the suffering, and paved the way to an easy and Christian death. It also prolonged life from Friday night till Monday morning following, thus allowing the children to come long distances to see their mother die. More than this, the homœopathic remedy restored consciousness so that the children and grandchildren could receive an intelligent word at the parting, and thus be blessed through life with a pleasant remembrance. Homœopathy, the boon of the ever-beneficent Creator to suffering humanity, the law by which pain is subdued, suffering mitigated, and death made easy.

Further comment is unnecessary. Those acquainted with the disease can readily see the action of the remedy, or remedies, in relieving distress and suffering. Possibly a more skilled prescriber might have saved the life, but I rest satisfied with the perfect and permanent euthanasia, fully believing that none but a true homœopathician could have accomplished that end in such a case. The triumph of Homœopathy was as great in this instance as though life had been saved, for in incurable disease the only object can be euthanasia, and that only is obtainable by the aid of our law of cure, the divine law of Homœopathy.

